# Gluing life together. Computer simulation in the life sciences: an introduction

**DOI:** 10.1007/s40656-018-0235-9

**Published:** 2018-11-22

**Authors:** Janina Wellmann

**Affiliations:** mecs – DFG-Kollegforschergruppe, Medienkulturen der Computersimulation, Leuphana Universität Lüneburg, Universitätsallee 1, 21335 Lüneburg, Germany

## Abstract

Over the course of the last three decades, computer simulations have become a major tool of doing science and engaging with the world, not least in an effort to predict and intervene in a future to come. Born in the context of the Second World War and the discipline of physics, simulations have long spread into most diverse fields of enquiry and technological application. This paper introduces a topical collection focussing on simulations in the life sciences. Echoing the current state of tinkering, fast developments, segmentation of knowledge and interdisciplinary collaboration, and in an effort to bridge the science-humanities divide, the contributors to this collection come from multiple disciplinary backgrounds, including information studies, cognitive sciences, philosophy and biology. The ambiguous character of simulations, their cutting across scientific disciplines, analysis and prediction, understanding and doing, gave rise to their success in contemporary life sciences and has been the object of much scientific debate. One of the main aims of this topical collection, by contrast, is to call into question the assumption of an obvious use and easy transfer of methods between fields of knowledge as diverse as, e.g. physics and biology. The collection presents historical case studies from various biological sub-fields. The articles study how simulations are used and the ways they contribute specifically to our understanding of life. Taking up Sergio Sismondo’s description of simulations as “compromises” and “glue”, they also critically engage with the question of what exactly the life sciences have been gluing together over the last two decades.

## Introduction

Computer simulations are ubiquitous in twenty-first century science, technology, and our everyday encounters with cars, planes or traffic control systems. In recent years, they have been the object of intense study and reflection, as developments proceed rapidly and with increasing diversity, and as the technology is applied to new fields.^1^ However, computer simulations first entered the scientific stage in physics in the context of the Second World War and the Manhattan Project (Fox Keller 2003; Galison 1997). Consequently, much of the historical and philosophical reflection on the epistemology of simulations has revolved around the specific scientific problems and questions faced within the discipline of physics, the twentieth century’s pre-eminent science. Of pivotal interest, in particular among philosophers of science, has been the question of how to relate the ways of doing science introduced by simulations to the traditional categories of theory, observation and experiment (see the now-classical studies of Gramelsberger 2011; Humphreys 2004; Winsberg 2010).

But with simulations being applied by an increasing number and variety of scientific disciplines, ranging from the social to the human and the life sciences, the epistemological framing of simulations has widened (see, for example, the recent overviews in Grüne-Yanoff and Weirich 2010; Lenhard 2016; Parker 2014; Winsberg 2017). Modelling, imaging and material practices have come into focus, and hence the role of simulations as models and mediators, with the computer operating as a dry lab and performing experiments in silico, thus bridging theory and experimentation (Humphreys and Imbert 2012; Knuuttila et al. 2006; Lenhard 2007; Morgan and Morrison 1999; Sismondo and Gissis 1999). By transgressing science, simulations have also become actors in the world. They draw together “webs of activities”. In constant change, they are “unfolding scientific objects”, whose advantage lies precisely in co-evolving within a net of various kinds of activities, encompassing scientific tinkering as well as political activism, and allowing the integration of “heterogeneous bodies of knowledge” (Knuuttila et al. 2006, quotes p. 6–7). To underline the epistemic specificity of computer simulations arising from the computer as a novel medium of doing research, Gabriele Gramelsberger has borrowed the notion of a “symbolic form” for computer simulations from Ernst Cassirer’s philosophy (Gramelsberger 2010).

## Simulation in the life sciences

The present collection, in contrast, focuses exclusively on the life sciences. In this field, computer simulations are relatively new, coming to the fore at the turn of the twenty-first century, and making a massive impact. The rise of systems and synthetic biology is one of the reasons for the surge of simulation approaches in the life sciences. Systems biology attempts to study life differently from the twentieth-century science of biology, by examining it computationally and in all its complexity, integrating information from the micro-level of molecules to the whole organism and its environment (e.g. Boogerd et al. 2007; Kitano 2002; Schneider 2013; Wake 2008). Investigating the dynamics of biological systems and breaking new grounds to predict their behaviour, the life sciences have seen fundamental changes in perspective, methodology, and understanding over the last half-century. This has prompted various attempts to refer to the present era as postgenomics (Reardon 2017; Richardson and Stevens 2015) and to frame the life sciences as “science without laws” (Creager et al. 2007) or “data-centric” (Leonelli 2012, 2016), working with “growing explanations” (Wise 2004) and “modelling from above” (Fox Keller 2003, p. 209) to account for the fact that much of biology, unlike physics, does not rely on theory.

In the life sciences, we find the whole range of common simulation techniques such as Monte Carlo simulations, agent-based modelling, cellular automata, finite-element simulations or artificial neural networks, to name only a few. Typically, simulations are used to guide the design of an experiment, explore the range of parameters, test hypotheses, and fit an experiment to the optimal use of material and intellectual resources. Arguably, one of the biggest advantages of simulation approaches is the change in perspective on the living world: imitating “one process by another process” (Hartmann 1996, p. 77), simulations enforce the study of the biological world in its temporal dimension. They frame the organism as a temporal being, unfolding and processing information in time, and being in continuous flux. Consequently, the use of computer simulations in the life sciences may easily be understood and may prove to be an example of the spreading of technology, once it is available, into new fields and enquiries. As has become clear from the trajectory of simulations in science and technology, the “hybridization” of disciplines and “cross-disciplinary traffic of technical innovations” (Fox Keller 2003, p. 199) is one of the vital characteristics of simulation approaches. For biology, this is undoubtedly the case, and this accounts for many of the recent transformations in the discipline. And yet, one of the central aims of this topical collection is to call into question the assumption of an obvious use and easy transfer of methods between fields of knowledge as diverse as physics and biology, for example.

To start with, one might scrutinise whether, and to what extent, physics and biology share the same epistemological interests. Working in a long-term research collaboration at the Weizmann Institute of Science in Israel, the computer scientist David Harel and the immunologist Irun Cohen voiced observations on their respective disciplines and transdisciplinary work. Physics, as they put it, is interested in being; biology, in contrast, in becoming. Biology is about the cursory details, the messy inconsistencies of life: While the elephant and the nematode *C. elegans* abide by the same laws of physics and chemistry, biology cannot be reduced to fundamental laws only but has to “attend to the fleeting details” to explain the differences in the forms of life (Cohen and Harel 2009, p. 2). At the same time, simulations have been credited with allowing scientists to “model from above” (Fox Keller 2003, p. 209) and put forth a “trial theory” (Fox Keller 2003, p. 206) for phenomena where there is no generally accepted theory in biology. Clarification is needed here, however, as to what exactly theory is referring to in the context of the life sciences, whether it is limited to mathematical formalization or addresses the more general foundations of biology (Laubichler and Müller 2007). But instead of entering contested territory, the answer to this epistemological question is being shifted more and more frequently to the technological domain. For many systems biologists, the new engineering vision of synthetic biology “follows naturally”^2^ from the ambition to examine and predict the complex behaviour of living systems. Constructing life, or, rather, bits and pieces of it, stands in for scientific analysis and understanding (see Gramelsberger et al. 2013; Roosth 2017).

In addition, doing simulations means using software. In a recent review article, Johannes Lenhard advocates computer simulations as a “combinatorial style of reasoning.” Lenhard argues that this kind of combinatorial reasoning creates “new similarities” cutting across established disciplines. He observes that, “in terms of theory”, disciplines such as biology and physics “look quite different. In terms of simulation modelling, however, they look quite comparable” (Lenhard 2016, p. 734). He likewise notes that simulation modelling often practically boils down to the traveling of “software packages” from one field to the other, thus contributing to an increasing gap “between model developers and model users” (Lenhard 2016, p. 734). But traveling software not only plays out in the drift between the reproducing and the understanding of biological phenomena but also in the imaging, or “animation” of life. Visual representation is fundamental for the processing of data, the analysis of simulation results, and display of complex organic features. Among the technological advancements that most deeply affect the course of twenty-first century life sciences is live-cell imaging (Landecker 2012; Lippincott-Schwartz et al. 2001; Miyawaki et al. 2003; Papkovsky 2010 ). Live-cell imaging enables scientists to image living organisms over time and under live conditions. Now, experimenting upon the living body, building models and imaging organic processes as they proceed in time are performed in unison. What kind of visual imagery of the living world, however, do computer animations create? And how does the use of a number of available software packages affect the suitability of simulations to capture the fleeting details of life? (see Carusi 2011; Carusi et al. 2015; Coopmans et al. 2014; Wellmann 2017).

Finally, modelling in computational biology implies a mathematical approach to life (Fox Keller 2002; Gramelsberger 2010). While there is considerable enthusiasm about “an explosive synergy” (Cohen 2004, p. 2017) between mathematics and biology for the benefit of both fields, and as regards the relationship of mathematics and theory building in biology, the equation of mathematical and simulation modelling raises epistemological concerns. As Gramelsberger has underlined, a simulation is not an identical but an “entirely new representation of the mathematical model from the perspective of its quantitative computation”. Rather than a model, a simulation, thus, is “a calculation rule for a specific mathematical model” (Gramelsberger 2010, p. 241). But are mathematical and simulation models comparable or rather incompatible in the attempt, for example, to capture the temporal dimension of biological phenomena, in particular, when it comes to understanding continuities or discontinuities, variation or iteration, linear or rhythmic patterns? These differentiations are of particular relevance for many biological phenomena. But rather than being critically addressed, the passage from the biological to the mathematical, to the computational, is more often than not performed smoothly.

In an early article on models and simulations, Sergio Sismondo described the ambiguous character of computer simulations as “compromises: they must simultaneously look like theory—because they have to explain, predict, and give structure—and like practical knowledge—because they have to connect to real-world features”. He pointedly speaks of simulations as “glue” (Sismondo 1999, p. 258). Twenty years later, the time has come to ask what exactly the life sciences have been gluing together over the last two decades. Are simulations built to capture the fleeting details of life? What images of life do their visual displays paint? And what are the compromises between the biological, mathematical, and computational that simulations entail? Indeed, are we still aware of what we are gluing together?

## Contributions to the topical collection

In view of the actual simulation practices in laboratories and the wide range of divergent topics, multiple objects, and behaviours being simulated, it seems promising to look into specific cases. That requires us to look at the messy details of everyday science in the making: What is claimed, what is actually done? What assumptions go into certain simulations and why? And what does that mean for our understanding of life? In this collection, contributors from the sciences and humanities engage in an interdisciplinary dialogue to tackle the complexities of today’s scientific endeavours. The authors of this collection examine computer simulations in fields of twenty-first century life sciences as diverse as evolution and cell biology; they approach these fields with equally diverse questions and methodological concerns, such as visualization, modelling and embodiment; and they have been trained in or have moved between various disciplines, such as information studies, cognitive sciences, philosophy, and biology. To highlight the specificities of simulation approaches in the world of the living is a major motivation for this collection, and the current state of tinkering, fast developments, disciplinary collaboration, segmentation of knowledge and unpredictable pathways in the life sciences is reflected in the diversity of the research presented here. At the same time, all the contributions, albeit in different, thought-provoking ways, are an invitation to reflect upon these developments critically.

Christopher Kelty, an information scientist trained in science studies, addresses the field of evolutionary biology, more precisely kin selection and Hamilton’s rule, which asserts that favouring an organism’s close relatives rather than the organism’s own reproductive success can be evolutionarily beneficial. He examines experimental attempts in “artificial evolution” (Kelty 2018, p. 2) to quantitatively test Hamilton’s rule both with physical and simulated robots. Introducing the literal and metaphorical figure of the robot—the robot as an actual actor in the experimental biological set-up and as a mechanical/digital copy of life—allows Kelty to discuss the experiment in terms of the fruitful distinction of ‘simulating something’ and ‘participating in’ something: do robots simulate evolution and life or do they participate in them? This distinction shifts the discussion of simulations away from clear-cut dichotomies to a more differentiated “scale of situatedness” (Kelty 2018, p. 3). Discussing robots with Deleuze as copy and simulacra, Kelty finally opens the discussion to the question of if and how, in the twenty-first century, we can distinguish between life and the machine, their shifting boundaries and our sense of control over either of them.

From the level of the whole organism and the grand theory of evolution the philosopher of science and media, Gabriele Gramelsberger, directs our attention to the inside of the body and shifts the perspective to the level of cells. Cells quickly became a topic of major enquiry as building blocks of life after Matthias Schleiden and Theodor Schwann formulated their cell theory in the middle of the nineteenth century. At the beginning of the twentieth century the invention of continuous culture techniques, a method that allows to manipulate the growth patterns of cells in culture, introduced a new dimension of mathematical modelling of cell processes. Gramelsberger studies the development of continuous culture techniques with a focus on Jacques Monod’s work in the 1950s and argues that, in this case, the experimental system functioned as a simulator of standardized growth processes, which were translated into a mathematical model. She explores how life processes were made to fit mathematical models (and vice versa) through the use and design of particular experimental systems. She thus highlights an interdependency between experimentation and mathematical modelling that is usually overlooked: the simulation does not run a mathematical model on the computer; rather, the experiment, as a simulation to obtain quantitative data, is itself heavily constrained by the model. In the experimental set-up, Gramelsberger holds, mathematics becomes material.

In Gramelsberger’s account, the development of modern biological lab science follows an ever-increasing automation of experimental procedures, to the degree that experiments become “self-controlled cybernetic simulators” (Gramelsberger 2018, p. 4). Nancy Nersessian, a cognitive scientist trained in physics, and Miles MacLeod, a philosopher of science trained in applied mathematics, shift the perspective by including the modellers in the simulations: they study the systems being modelled, and the people modelling them, as a coupled system. The authors draw on ethnographic research undertaken in a number of systems biology laboratories over several years. In these labs, researchers from various disciplines, including engineers, biologists, and mathematicians, engaged in building models for various gene regulatory, metabolic and cell signalling networks. Looking into the details of the frequently painstaking process of model building, Nersessian and MacLeod explore the cognitive dimension of model building and simulation. In modelling, not only different disciplines and styles of reasoning meet, but also the person of the scientist with her lab instruments and technologies. The authors argue that the modeller and the model form a “cognitive coupled system” (MacLeod and Nersessian 2018, p. 4). In these coupled systems, the scientists mentally simulate as well: they actively engage in cognitive simulations in order to understand and predict a limited number of aspects and outcomes of the systems they study, as well as to test hypotheses and make actual choices for their models. The limited human cognitive ability to tackle complexity, therefore, not only informs, but effectively limits, simulation and model building. With the growing complexity of models and systems, the authors expect “cognitive factors to play an increasingly prominent role” rather than a decreasing one (MacLeod and Nersessian 2018, p. 25).

Thomas Washausen, Wolfgang Knabe and their collaborators, a team of experimental biologists and computer scientists, study the phenomenon of programmed cell death (apoptosis) in the process of neurulation during early embryogenesis of a tree shrew species. Using three-dimensional simulation and live-cell imaging tools, the authors capture the spatiotemporal patterns of cell death flowing through the spinal cord of the embryo in rhythmical waves. Simulating the process enables them to give a nuanced picture of the different temporal dynamics involved in the process, in particular static patterns and dynamic rhythms, a distinction that is of prime importance for understanding developmental events and reconciling hitherto inconsistent experimental findings (Washausen et al. 2018). The work of the biologists provides important insights into the use and adaptation of imaging techniques for practical experimental and simulation work and decision-making.

The last contribution in this topical collection, by Janina Wellmann, complements the inside view into contemporary practices with a broadly construed historical case study of imaging technologies in embryogenesis. Wellmann examines recent live-cell imaging technologies to capture cell movement against the backdrop of various imaging, modelling and simulation approaches to researching early embryogenesis throughout the twentieth century. She shows that the understanding of morphogenesis has faced a major dilemma right from its early investigation: representing form and understanding movement are mutually exclusive, as are understanding form and representing movement. Providing a solution to this dilemma only on face value, she argues, live-cell animations are visual simulations that obscure rather than advance the understanding of morphogenesis (Wellmann 2018).

In sum, this collection presents a broad spectrum of approaches to simulation in contemporary life sciences. Although they examine a range of fields and practices, all the articles share a common concern. They shed light on those facets of simulation which, in much of the contemporary discussion, are considered to be precisely what simulations are built to dispose of: embodiment and participation (Kelty), the human factor (MacLeod and Nersessian), biological temporalities (Washausen et al.), the materiality of simulations to make life fit to mathematics rather than considering simulations as epistemic tools (Gramelsberger), and the paradoxes of their visual instantiations (Wellmann).

## Footnotes

1 To give an impression, see for the social sciences, Suleiman et al. (2000); archaeology, Wurzer et al. (2015); surgery, Dasgupta et al. (2013), Prentice (2005); medicine, Edwards et al. (2010); heritage studies, Ch'ng et al. (2013); and for various applications in the humanities, or the organization of our social world, Turkle et al. (2009).

2 Pamela A. Silver, founding member of the Department of Systems Biology at Harvard Medical School, https://harvardmagazine.com/2011/09/biology-in-this-century (last accessed November 10, 2017).
